# Influence of Atmospheric Phosphorus and Nitrogen Sedimentation on Water Quality in the Middle Route Project of the South-to-North Water Diversion in Henan Province

**DOI:** 10.3390/ijerph192114346

**Published:** 2022-11-02

**Authors:** Yunlin Qiu, Yun Zhang, Pengcheng Lan, Han Liu, Hongtian Wang, Wanping Wang, Peng Zhao, Yuying Li

**Affiliations:** 1 College of Oceanic and Atmospheric Sciences, Ocean University of China, Qingdao 266100, China; 2 International Joint Laboratory of Watershed Ecological Security and Collaborative Innovation Center of Water Security for Water Source Region of Middle Route Project of South-North Water Diversion in Henan Province, College of Water Resource and Environment Engineering, Nanyang Normal University, Nanyang 473061, China; 3 Nanyang Management Office of Qushou Filiale of Middle Route Project of South-North Water Division, Nanyang 473013, China

**Keywords:** dry and wet deposition, atmospheric nitrogen and phosphorus, water quality, the Middle Route Project of South-to-North Water Diversion, ecological impact

## Abstract

In order to understand the potential effects of atmospheric nitrogen and phosphorus deposition on the water quality of the Middle Route Project of the South-to-North Water Diversion Project, samples of dry and wet deposition of atmospheric nitrogen and phosphorus, meteorological factors, and water quality factors were analyzed out to investigate in the Middle Route Project of the South-to-North Water Diversion in Henan Province from October 2018 to October 2020. The variation characteristics of atmospheric nitrogen and phosphorus deposition with time in the Henan section of the main canal are revealed, and the influence of atmospheric dry and wet deposition on the water quality of the middle line is discussed. It was found that the total nitrogen (TN) sedimentation flux has obvious seasonal variation, which was consistent with the variation trend of rainfall, and increased with the increase of rainfall. Nitrogen and phosphorus deposition was significantly correlated with water factors. The effects of meteorological factors and nitrogen and phosphorus deposition on water quality variation reached 18%. The contribution rate and ecological impact of atmospheric nitrogen and phosphorus deposition on water pollution of main channels will be increasing, which needs to be paid enough attention to.

## 1. Introduction

The Middle Route Project (MRP) of the South-to-North Water Diversion Project (SNWD) in China is the world’s longest cross-basin water diversion project, with a total length of 1432 km. Since the launch of the water diversion project in 2014, more than 44 billion cubic meters of water have been transferred to the north, directly benefiting 140 million people and greatly alleviating the water shortage in the north. The south-north water diversion project effectively improves the ecological environment of filling water areas, but as a closed water ecosystem, no person or animal can enter the channel except channel managers. Except for rainfall, no other water can enter the canal. Only trees can be planted within 30 m on both sides of the passage, and agricultural and industrial activities are not allowed. Therefore, the water quality in the channel is less affected by human activities, but cannot avoid the impact of the atmospheric environment; excessive nitrogen (N) and phosphorus (P) nutrients deposition can reduce ecosystem biodiversity, eutrophication and acidification and a series of negative ecological effects [1], directly affect the water quality safety of the MRP of the SNWD. Therefore, the observations, sources and impacts of atmospheric dry and wet deposition along the MRP of the SNWD are of great significance to the scientific formulation of water quality management policies, in-depth understanding and exploration of water quality.

Nitrogen, phosphorus and other elements in the atmosphere settle on land or water in certain ways, which become an important way of the biogeochemical cycle and play a very important role in natural ecosystems such as ocean and forest [2]. Since entering the stage of rapid development of industrialization, human activities such as massive fossil fuel consumption and fertilizer application have led to a rapid increase in the deposition of atmospheric elements such as N and P [3], resulting in changes in the N cycle and a significant impact on the ecosystem. Studies have shown that atmospheric deposition is an important way for nitrogen and phosphorus nutrients to be imported into aquatic ecosystems and an important source of nutrients for aquatic organisms [4]. A large number of nitrogenous compounds caused by atmospheric subsidence have an important effect on the growth and population structure of phytoplankton [5,6,7]. It has also been pointed out that atmospheric N deposition has become an important source of eutrophication in some estuaries, coastal waters and freshwater bodies [8,9,10].

At present, studies on atmospheric deposition mainly focus on the N cycle [4] and its impact on marine, soil, forest, and grassland ecosystems [11,12,13]. Although early studies believed that the contribution rate of atmospheric N and P deposition to the pollution load of natural ecosystems was relatively low [14], more and more studies showed that excessive nitrogen and phosphorus deposition would significantly affect the structure and function of natural ecosystems such as forest, ocean, farmland and grassland [15].

Although the amount of atmospheric deposition of N and P was less than that of other sources leading to water eutrophication, atmospheric deposition of N and P still contributes to the concentration of N and P in water. It is generally believed that when the amount of inorganic nitrogen deposition is within a certain range, most of the nitrogen is retained in the ecosystem, but 25 kg/hm^2^·a is generally considered to be a critical point, beyond which the Nitrogen saturation occurs. Experiments have also shown that when the nitrogen deposition is in the range of 10–15 kg/hm^2^·a, the nitrogen output of the forest ecosystem will increase significantly. The amount of nitrogen deposition in some areas of my country has exceeded twice the critical point, and in the future, the amount of nitrogen deposition in my country may continue to increase, which will inevitably affect the function of forest ecosystems. Our country has become one of the three most concentrated areas of nitrogen deposition in the world (the United States, northern Europe and China) and has received extensive attention from the international community. Excess N deposition will accelerate soil acidification and water eutrophication [1]. Zheng Baohai found that the TN concentration in Danjiangkou Reservoir was 0.97 mg/L in 2017, 1.38 mg/L in 2018, and the TN concentration in Danjiangkou Reservoir was relatively high, according to the study on the characteristics and driving factors of the community structure of planktonic eukaryotes in Danjiangkou Reservoir [16]. Chen Zhaojin studied the composition of the bacterioplankton community and its influencing factors on the MRP in Henan Province. He found that the TN concentration in the canal was 0.87~1.08 mg/L in 2017, and the canal trophic state index (EI) was 47.5~52.5, indicating that the canal’s trophic state ranged from moderate to slightly eutrophic [17]. Therefore, understanding the deposition characteristics of atmospheric N and P and estimating the deposition flux of atmospheric N and P are of great significance for the prevention and control of N and P pollution in water bodies, especially for the artificial channels with high N elements, such as the MRP of the SNWD. Understanding the deposition flux of N and P was of practical significance for the prevention and control of eutrophication in water bodies.

The South-to-North Water Diversion Project has alleviated the serious shortage of water resources in the north, thereby increasing the carrying capacity of water resources, improving the efficiency of resource allocation, and providing a guarantee for the economic development of the North. Nitrogen and phosphorus deposition has a great impact on the chemical properties of water in the South-to-North Water Diversion Project, which is related to the ecological development along the route and the quality of drinking water. However, previous studies on canal water quality mostly focused on plankton and water quality itself and rarely involved the impact of atmospheric N and P deposition on canal water quality. This paper studies the amount of N and P deposition in the canal of Henan Province and discusses for the first time the influencing factors of N and P deposition in the atmosphere of Henan Province and the influence of N and P deposition on the canal water quality. The following questions are mainly studied: (1) Whether nitrogen and phosphorus deposition has seasonal changes. (2) The trend of nitrogen and phosphorus deposition and rainfall. (3) Changes in the weights of nitrogen and phosphorus deposition and rainfall on water quality. This study could provide a theoretical basis and technical support for the water quality safety of the South-to-North Water Diversion Project.

## 2. Research Area and Methods

### 2.1. Research Area

The Middle Route Project (MRP) of the South-to-North Water Diversion (SNWD) in China was located at 32°40′32″~36°05′36″ (N) and 111°37′08″~114°14′01″ (E). The study area spans the Yangtze River, Huaihe River, Yellow River and Haihe River basins. The average annual temperature was 10.5~16.7 °C from south to north, and the average annual precipitation was 407.7~1295.8 mm. The time of maximum precipitation was June to August, and the average annual sunshine was 1285.7~2292.9 h. Annual frost-free period 201~285 days.

**Figure 1:**
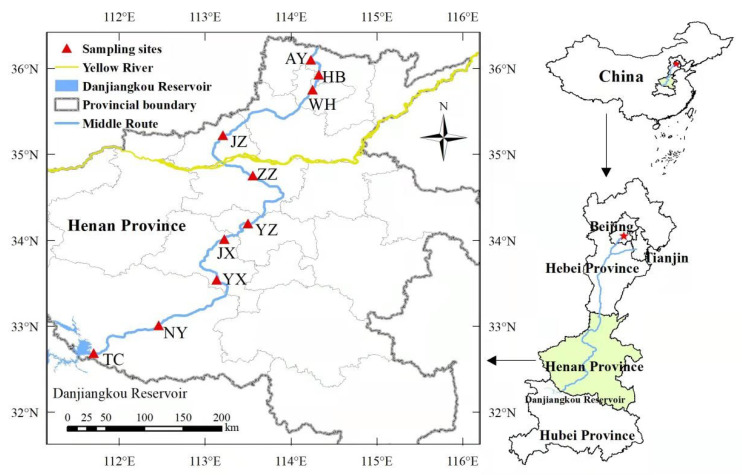
Locations of sampling in the MRP of the SNWD.

According to the on-site inspection and demonstration, for Table 1 and Figure 1, 10 ecological monitoring stations were identified, which were located on the right bank of the Taocha Dam (TC), Nanyang Management Office (NY), Yexian (YX), Yuxian (JX), Yuzhou (YZ) Zhengzhou (ZZ), Jiaozuo (JZ), Weihui (WH), Hebi (HB) and Anyang (AY). Since rainfall has a significant impact on atmospheric N and P deposition fluxes, this study will explore the characteristics of N and P deposition fluxes in dry and wet seasons. As the research area belongs to the temperate continental climate, rainfall is concentrated in spring and summer, and precipitation was less in autumn and winter, so it was divided into dry and wet seasons to explore the N and P deposition flux change characteristics of monitoring points. The wet season is from May to October, and the dry season is from November to April of the following year. Both the wet and dry seasons last for 6 months.

### 2.2. Research Methodology

The PC-8Z Atmospheric Grid Monitoring System was connected with the Sunshine Smart cloud platform to form Atmospheric Grid Monitoring and obtain meteorological factors. The design module can automatically monitor the ambient temperature (AT), ambient humidity (AH), wind speed (WS), atmospheric pressure (AP), fine particulate matter PM_2.5_ and inhalable particulate matter PM_10_. All monitored data was automatically uploaded to the cloud platform (website: www.jzygzhy.com, Sunshine Meteorology Tech. Co., Ltd., Liaoning, China).

In this study, GH-200 automatic sampler for precipitation and dust removal (Genstar, Qingdao Genstar Electronic Technology Co., Ltd, Qingdao, China) was used to collect dry and wet deposition samples. The sampling method of dry sedimentation samples refers to the collection method of dust in the “Environmental Monitoring and Analysis Method and Air and Waste Gas Monitoring and Analysis Method.” Samples were collected once at the end of each month, and the dust attached to the inner wall of the collection bucket was washed with deionized water, collected in polyethylene plastic bottles, and then filtered with 0.45 µm water microporous filter membrane, and returned to the laboratory for testing as soon as possible.

The collection and preservation methods of wet deposition samples refer to “Collection and Preservation of Precipitation Samples” (GB 135.802-92). Collect after each rainfall or snowfall, and collect 1–3 rain samples depending on the rainfall or snowfall. The whole process of each precipitation was taken as a rainfall sample (from the beginning to the end of precipitation). If there are several precipitation processes in a day, they can be merged into one sample for collection. If it rains for several consecutive days, the precipitation from 8:00 am to 8:00 am the next day can be collected; that is, the 24-h precipitation sample can be taken as a sample. If the amount of sample collected in one rainfall cannot reach the amount required for the test (100 mL), the samples collected before and after are mixed into one sample. Then the samples are sent back to the lab for testing.

Water quality indicators are determined in accordance with the “China Surface Water Environmental Quality Standard” (GB3838-2002). Dissolved oxygen (DO), pH, electrical conductivity (Cond), and oxidation-reduction potential (ORP) were measured in the field with a YSI 6920 (YSI Inc., Yellow Springs, OH, USA). Two-liter water samples were collected using a sterilized column water extracto and sent to the laboratory for chemical analysis within 24 h. Permanganate index (COD_Mn_) was determined using the potassium permanganate digestion titration method. Chlorophyll a (Chla) concentration was spectrophotometrically measured after extraction in 90% ethanol. Total phosphorus (TP) was determined with acidified molybdate to form reduced phosphomolybdenum blue, which was spectrophotometrically measured. Total nitrogen (TN) was assayed using alkaline persulfate digestion and UV spectrophotometry, while ammonia nitrogen (NH_4_^+^-N) and nitrate-nitrogen (NO_3_^−^-N) were measured by the spectrophotometric method using Nessler’s reagent and thymol spectrophotometry, respectively. Total organic carbon (TOC) was determined using a TOC analyzer (Multi N/C 3100, Analytik Jena Ltd., Jena, Germany) with regards to the environmental protection standard “Determination of water quality of total organic carbon combustion oxidation—non-dispersive infrared absorption method” (HJ501-2009).

### 2.3. Data Analysis

Formula (1) shows that the atmospheric dry sedimentation flux was estimated using atmospheric concentration and sedimentation velocity (*F_d_^,^* kg/hm^2^). The sedimentation rate of coarse particles is 2 cm/s:

(1) $$F_{d^{\prime }}=\sum_{n=1}^{m}\left(C_{n}^{c}*V_{d}^{c}+C_{n}^{f}*V_{d}^{f}\right)$$

In Formula (1), *F_d_*, the dry sedimentation flux (kg/hm^2^), *C^c^_n_* represents the monthly average concentration of particulate matter n coarse mode (μg/m^3^), *C^f^_n_* indicates the monthly average concentration of particulate matter n fine modalities (μg/m^3^), *V^c^_d_* and *V^f^_d_* for the monthly sedimentation rate of particulate matter (cm/s).

The atmospheric wet sedimentation flux was to take the weighted average of each precipitation component concentration and precipitation as the average concentration of the components contained in the monthly precipitation, and finally, the atmospheric wet sedimentation flux is obtained from the average monthly concentration and precipitation (*F_w_*, kg/km^2^):

(2) $$F_{w}=\sum_{a=1}^{n}\left(C_{i}*P_{i}\right)*S$$

In Formula (2), *F_w_* is the lunar atmospheric wet sedimentation flux (kg/km^2^) and *C_i_* is the average concentration of the constituent compounds (mg/L):

(3) $$C_{i}=\sum_{a=1}^{n}\left(C_{a}*P_{a}\right)/\sum_{a=1}^{n}*\;P_{a}$$

*P_i_* is monthly precipitation (mm) and S is the area of the region. In Formula (3), *C_a_* is the concentration of the constituent compounds contained in each precipitation (mg/L), *P_a_* is precipitation (mm), and *C_i_* is the average concentration of the constituent compounds (mg/L).

Formula (1) is used to calculate and estimate atmospheric concentration and sedimentation velocity. Formula (2) is used to calculate the average concentration of the constituent compounds. Formula (3) is used to calculate the lunar atmospheric wet sedimentation flux.

All data were processed using Excel 2019 (Microsoft Corporation, Redmond, WA, USA). Detrended correspondence analysis results show that the maximum gradient length is less than 3. Therefore, the next step is redundancy analysis (RDA). The relationship between nitrogen and phosphorus deposition and physical and chemical parameters of water quality was analyzed by the Pearson analysis method. Using the “pure” package in R, variance partition analysis (VPA) and variance decomposition analysis method, the explanation rate of atmospheric nitrogen and phosphorus deposition and atmospheric environmental factors on water quality in Henan Canal was calculated. ArcGIS 10.7 (ESRI, Redlands, CA, USA) was used as a spatial analysis tool to visualize the sampling stations of the canal in Henan Province. SPSS 25 (IBM SPSS Inc., Chicago, IL, USA) was used to calculate the mean values and SD of meteorological factors and water quality factors.

## 3. Results

**Figure 2:**
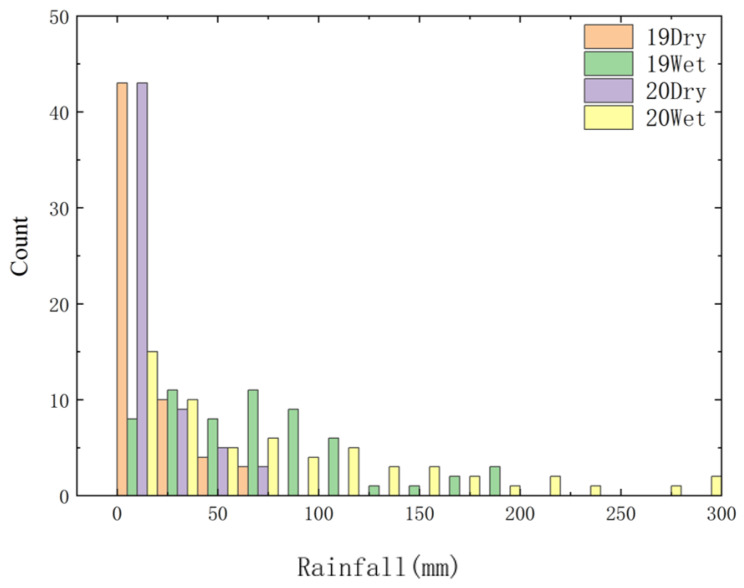
The average monthly rainfall of the canal in Henan Province.

The average monthly rainfall in 2019 was 11.46 mm in the dry season and 68.91 mm in the wet season. The average monthly rainfall in 2020 was 18.24 mm in the dry season and 96.87 mm in the wet season. Average monthly rainfall was slightly higher in 2020 than that in 2019 (Figure 2).

**Figure 3:**
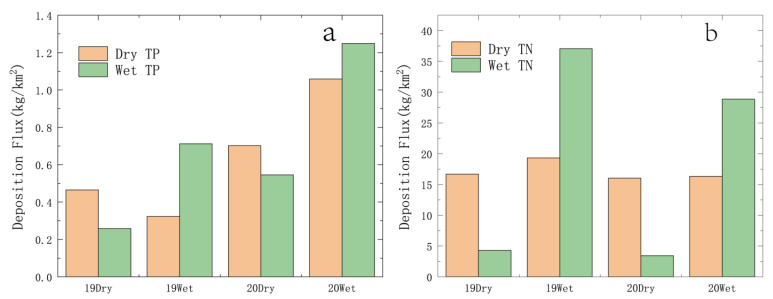
Atmospheric nitrogen and phosphorus deposition fluxes in dry and wet seasons. (**a**) Dry and Wet TP deposition; (**b**) Dry and Wet TN deposition.

Figure 3 shows the changes in TP and TN deposition from October 2018 to October 2020; TP deposition in the southern section of the canal has obvious changes with time. During the monitoring period, TP deposition flux showed a gradually increasing trend and reached the highest value of 1.25 (kg/km^2^) in the wet season of 2020. The TN deposition flux changed relatively little with time, and the dry TN deposition flux fluctuated little and was stable at about 17 kg/km^2^ during the survey period. The variation trend of wet TN deposition flux is consistent with that of rainfall. With the increase in rainfall, TN deposition flux reaches a peak in the wet season. In terms of time, TP deposition flux has little change in the dry season and wet season, while TN deposition flux has a great difference in the dry season and wet season. On the whole, TN settlement in the wet season is higher than that in the dry season, while TP settlement has the same rule.

Henan Province has a temperate continental climate with four distinct seasons. The sun shines directly on the Northern hemisphere from March to September each year, so the temperature in the wet season is higher than that in the dry season. The atmospheric pressure is higher in the dry season than in the wet season. The ambient humidity in the wet season is higher than that in the dry season, which is the reason why the rainfall is concentrated in the wet season. During the study period, the wind speed of the canal in Henan province did not change greatly. Both PM_2.5_ and PM_10_ reached their lowest values during the wet season in 2020, which might be related to the significant decrease in human activities during the COVID-19 pandemic. During the investigation, all variables except TN were consistent with the environmental quality standards of Class I–II surface water quality (GB38382-2002). The mean and standard deviation (SD) of the water quality factors at the 10 sampling points were shown in Table 2. In all sampling stations, the spatial differences of COD_Mn_, TOC and TP were significant (*p* < 0.05), while the spatial differences of other physical and chemical factors were not significant.

In terms of time, Cond, ORP, DO, TP, COD_Mn_ and Chla fluctuated greatly with seasons, while other variables did not fluctuate significantly between seasons. Among them, AH, TOC, TP and NH_4_^+^-N had significant differences between seasons, while other indicators had no significant differences between seasons (Table 3).

**Figure 4:**
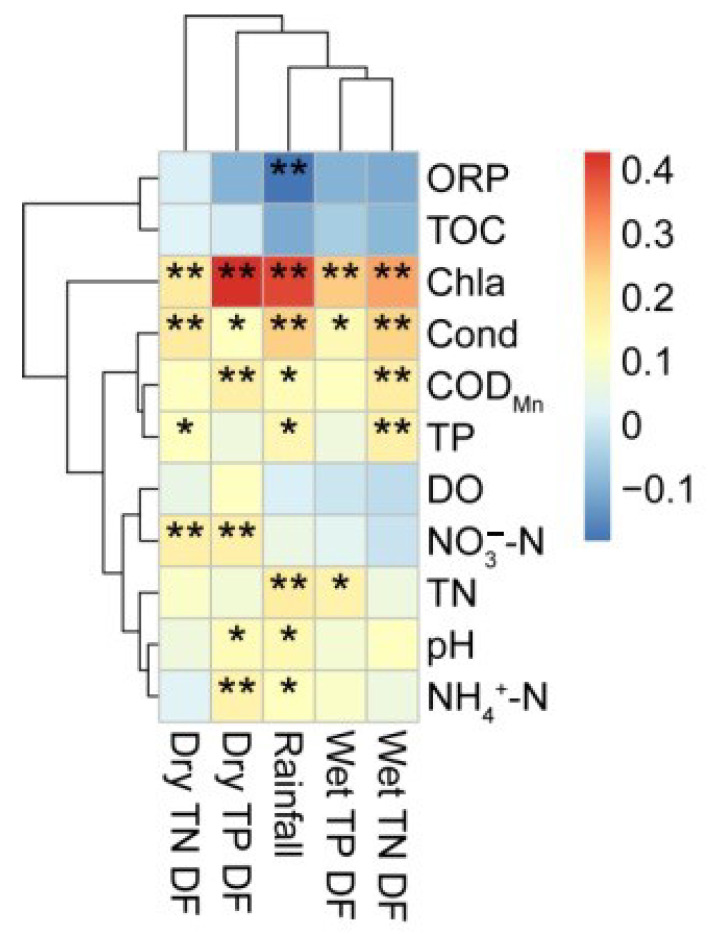
Relationship between atmospheric nitrogen and phosphorus deposition fluxes and rainfall and water quality factors. * means *p* < 0.05, ** means *p* < 0.01.

Figure 4 provides the relationship between atmospheric nitrogen and phosphorus deposition fluxes and rainfall and water quality factors. Pearson’s correlation analysis was used to determine the relationship between atmospheric deposition and water quality. The deposition flux of wet TN was significantly positively correlated with Chla, Cond, COD_Mn_ and TP at 0.01 level. Rainfall was positively correlated with Chla, Cond, COD_Mn_, TP, TN, pH, and NH_4_^+^-N and negatively correlated with ORP at 0.01 level. We can clearly find that the deposition flux of wet TP was positively correlated with Chla, Cond and TN. The deposition flux of dry TP was positively correlated with Chla, Cond, COD_Mn_, NH_4_^+^-N, NO_3_^−^-N and pH. The deposition flux of dry TN was positively correlated with Chla, Cond, TP and NO_3_^−^-N.

**Figure 5:**
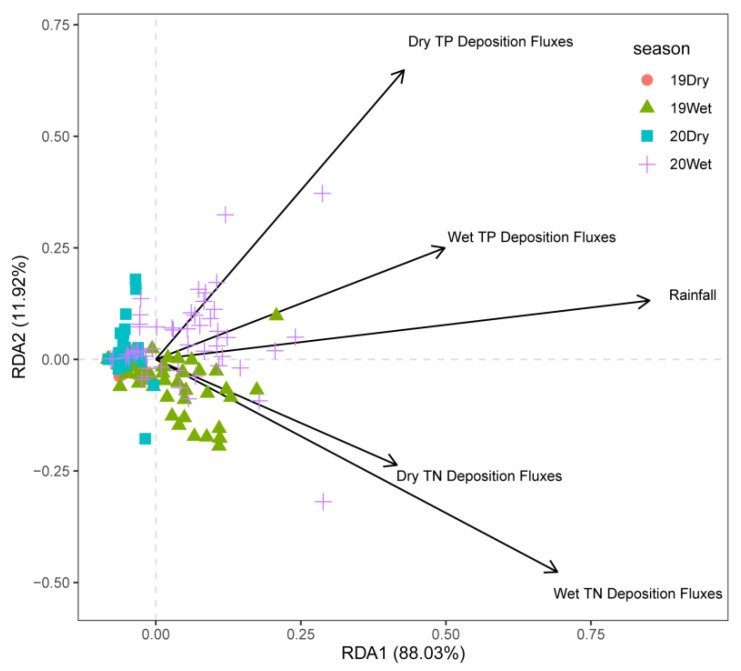
Redundancy analysis (RDA) of atmospheric nitrogen and phosphorus deposition and water quality factors in Henan Canal.

Detrended correspondence analysis shows that the maximum gradient lengths of the four axes are all less than 3. Therefore, we chose the RDA linear model, as shown in Figure 5. The RDA results are shown in Figure 5. The interpretation amount of the RDA1 axis and RDA2 axis was 88.03% and 11.92%, respectively. From the perspective of time, TN deposition flux had a significant impact on the water quality of the trunk canal in 2019, while TP deposition flux and rainfall had significant impacts on the water quality of the trunk canal in 2020. The dry TP deposition flux and wet TP deposition flux have a more significant impact on the water quality in the wet season in 2020, and the dry TN deposition flux and wet TN deposition flux have a more significant impact on the water quality in the wet season in 2019. The impact of rainfall on water quality was more pronounced during the wet season in 2019 and 2020.

**Figure 6:**
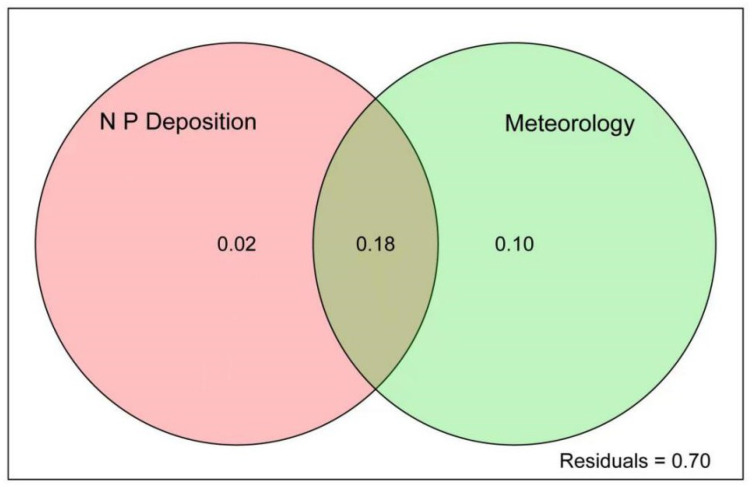
Variance decomposition of atmospheric N and P deposition and atmospheric environmental factors on water quality.

In order to clarify the relative influence of N and P deposition and atmospheric environment on the water quality of the canal in Henan province, VPA was used to calculate the explanatory rate of N and P deposition and atmospheric environment on the water quality of the MRP (Figure 6). The results showed that N and P deposition and atmospheric environment accounted for 2% and 10% of the water quality of the canal, and together accounted for 18% of the water quality of the canal, 70% of which could not be explained. It also shows that the atmospheric environment has a higher effect on the water quality of the canal than the effect of N and P deposition on the water quality of the canal. And the combined effect of N and P deposition and atmospheric environment on the water quality of the canal was also higher than the single effect of N and P deposition on the water quality of the canal.

## 4. Discussion

Atmospheric dry and wet deposition was affected by many factors in different seasons, different climatic regions and different ecological conditions [18]. The main factors were: source, rainfall, wind speed and direction, etc. Jia Junyan [19] found that there was a strong positive correlation between rainfall and N deposition in the southeast of Tibet, and the seasonal difference of N deposition was mainly related to the concentration and rainfall of various forms of N in precipitation in the corresponding period. It was understood that Henan Province belongs to the temperate continental climate, and rainfall was concentrated from May to October. Comparing the atmospheric N and P deposition with rainfall (Figure 2 and Figure 3), it is found that TN deposition flux is related to the variation trend of rainfall, while TP is the opposite. TN deposition flux increased with the increase in rainfall, while TP deposition gradually increased from the dry season of 2019 to the wet season of 2020. This is because N mainly exists in the gaseous form in the air and is easily dissolved in water during rainfall, while P in the air is easy to adhere to particles, which usually settle to the ground through gravity sedimentation and other forms. Therefore, the variation trend of rainfall is consistent with that of TN sedimentation flux, and this also shows that the rainfall has been polluted. The reason may be related to the agricultural activities in Henan Province. Studies have shown that in wet sedimentation, ammonium nitrogen mainly comes from the volatilization of ammonium nitrogen in soil, fertilizer and livestock manure, among which agricultural activities such as fertilization have a greater impact. However, Henan Province is a large agricultural province with a grain-sown area of more than 106,700 square kilometers. A large number of agricultural activities will undoubtedly lead to the increase of nitrogen content in precipitation. Wang Jiangfei [20] studied the dry and wet deposition of atmospheric N in the Hangjia-hu region and concluded that the ratio of dry and wet deposition was 1:1, and some domestic and foreign scholars found that the phosphorus sedimentation is given priority to with dry fall [21], with the conclusion of this study is not different. After studying nitrogen deposition in Daya bay, Huizhou, it was concluded that N deposition had seasonal variation [8], which was similar to the conclusion of this study. Correlation analysis showed that rainfall was also one of the important factors affecting the dry and wet deposition of N and P (Figure 5), and this is consistent with the research results on the composition, spatial pattern and influencing factors of atmospheric wet nitrogen deposition in China’s terrestrial ecosystems [22]. Early studies have shown that in the absence of rainfall, pollutants will be transported and spread in the atmosphere, continue to migrate to the ground, and constantly be absorbed by the underlying surface (land and water) [23].

The interval of rainfall is also one of the factors affecting N and P sedimentation. Through the study of nitrogen and phosphorus deposition input and influencing factors in the East Lake of Wuhan, it was found that the interval of rainfall could significantly affect the atmospheric N and P deposition load in the East Lake of Wuhan, and the main reason may be that the longer the interval of rainfall, the more dry deposition accumulated in the sampler, resulting in the increase of nitrogen and P deposition load [24]. In the wet season with abundant rainfall, N compounds such as ammonium salt and nitrate, which are easily soluble in water, accumulate through wet deposition, resulting in an increase in TN deposition in the wet season. In addition, with different organic matter and nitrogen and phosphorus in the standard state usually take the form of particles, phosphorus in the atmospheric circulation aerosol particle pollution in the air more easily in the process of transport by gravity settling, touch between particles and the function of settlement to the ground and leave air masses, mainly for the atmospheric circulation particles under the action of gravity settling, less affected by seasonal changes [25].

Hu Yang studied the spatio-temporal pattern of N and P wet deposition in the surrounding area of Taihu Lake and its influence on water quality and primary productivity. The results showed that NH_4_^+^-N in wet deposition mainly came from the volatilization of ammonium nitrogen in soil, fertilizer and livestock manure, and agricultural activities such as fertilization had a great influence on it. However, NO_3_^−^-N is mainly generated in two ways, one is formed by lightning strikes, and the other is generated by industrial and civil combustion fuels and automobile exhaust emissions [26,27]. All the sampling points set in this study were located around the city or beside traffic trunk roads, where industrial pollution and automobile exhaust emissions are relatively large, and agricultural production activities are frequent in the wet season, resulting in increased NH_4_^+^-N content of volatilization into the air. Therefore, TN deposition in the wet season is higher than that in the dry season. It is found that the seasonal variation pattern of atmospheric N deposition flux of the canal in Henan province was obviously different, and its seasonal variation trend is roughly the same as that of rainfall, showing a pattern of high in the wet season and low in the dry season. This pattern may be caused by the high rainfall (37.49% of the annual rainfall) and frequent rainfall from May to October in Henan Province and the frequent agricultural production activities in this period.

Existing research results show that N and P in lake water are mainly derived from exogenous input and endogenous release. The management and treatment of nitrogen and phosphorus mainly focus on point sources, non-point sources and endogenous sources [28], and less attention is paid to the influence of atmospheric N and P deposition. In recent years, many lakes have taken measures such as source control, pollution interception and ecological restoration to reduce and control pollution sources such as point sources, non-point sources and internal sources [24]. Although some treatment effects have been achieved, the contribution and influence of atmospheric N and P deposition have not been considered. In recent decades, the atmospheric deposition flux in China has shown a significant increasing trend. The national average annual N deposition flux increased from 13.2 kg/hm^2^·a to 21.1 kg/hm^2^·a, increasing by nearly 60% from the early 1980 s to the early 21 st century [6]. The study of Taihu Lake Basin also found that during the 10 years from 2002 to 2011, N wet deposition flux increased from 28.06 kg/hm^2^·a to 56.25 kg/hm^2^·a, P wet deposition flux increased from 3.14 kg/hm^2^·a to 13.64 kg/hm^2^·a, with growth rates of 112% and 77%, respectively [29]. The increase of dry and wet deposition of N and P and rainfall would lead to the increase of N, P, Chla and Cond concentrations in the water (Figure 4). Studies have shown that atmospheric deposition is an important way to import N and P nutrients into aquatic ecosystems and an important source of nutrients needed by aquatic organisms [4]. When the N deposition flux is within 25 × 10^2^ mg/m^2^, most of the nitrogen is retained in the ecosystem, but beyond this value, saturation will occur, and excess N deposition will accelerate the eutrophication of water [30]. However, the TN settlement flux in Henan province of the channel in the wet season exceeds this value (Figure 3), combined with the high TN concentration in Danjiangkou Reservoir and the channel of the middle line (Table 3) [16,23]; therefore, there is a risk of eutrophication in the water quality of the canal of the middle line. Nutrients are essential for the growth and reproduction of phytoplankton and are also important environmental factors affecting the seasonal variation of phytoplankton [31]. TN and TP are important factors affecting the cell density and community structure of phytoplankton. Studies have shown that the rapid uptake and utilization of N is the main reason for the massive proliferation of algae in early spring [32]. Barak Herut studied the atmospheric input of nitrogen and phosphorus to the Southeast Mediterranean: Sources, fluxes, and possible impact. It was found that specific rainfall or sandstorm events may still lead to measurable phytoplankton proliferation [33]. Our study also showed a significant positive correlation between N and P deposition fluxes and Chla, indicating that an increase in N and P would promote the growth of phytoplankton. According to our research results, the total annual settlement in the channel in Henan province was 3.80 × 10^4^ t, and the annual settlement of TN and TP was 10.75 t and 0.02 t, respectively. These nutrients provide the necessary conditions for the growth of phytoplankton in the trunk channels. The phytoplankton in the channel had a phenomenon of rapid biomass proliferation in a certain period of time, which became a potential ecological problem affecting the water quality of the transported water [34]. Yan Guanghan found that the change of Cond may be related to the eutrophication degree of the water body through the correlation analysis between chlorophyll concentration and environmental factors in Dongting Lake [35]. In this study, Cond concentration was significantly positively correlated with N and P deposition and rainfall, which is consistent with Yan’s findings.

Although the atmospheric deposition of N and P was less than that of other sources leading to the eutrophication of water, it still contributes to the concentration of N and P in water. VPA analysis showed that atmospheric N and P deposition and atmospheric environmental factors contributed 18% to the explanation of water quality (Figure 6). Although the contribution of N and P deposition to water quality is not high at present, according to the research results on the increase of atmospheric N and P deposition in the past two years, the atmospheric N and P deposition, especially the P deposition load, will increase greatly in the future. Therefore, under the condition that exogenous nitrogen and phosphorus input from point and non-point sources is gradually controlled, the contribution rate and ecological impact of atmospheric N and P deposition on water pollution of main channels will become larger and larger, which needs to be paid enough attention to.

The main canal of the SNWDP middle route is 1432 km long and runs across four provinces. The atmospheric N and P deposition showed an important influence on the water quality of the main channel, and the great length of the main channel in the middle line could further enhance the effect of atmospheric deposition. It was found that an important way of nutrient input to Chaohu Lake was the inflow river, which accounted for 90.18% of TN and 95.23% of TP [36]. In another study, one of the important reasons for the eutrophication of Dianchi lake is the pollutants in the rivers entering Dianchi Lake [37]. In the follow-up studies, the influence of atmospheric N and P deposition in other areas of the main canal can be explored as this study, and its influence on water quality of the final water supply can be comprehensively analyzed by combining the data in different main canal regions.

## 5. Conclusions

During the study period, the TN sedimentation flux had obvious seasonal variation, which was consistent with the variation trend of rainfall and increased with the increase of rainfall. There was no obvious seasonal variation of TP deposition flux, but it showed a gradually increasing trend during the study period. Atmospheric N and P deposition and rainfall had a significant influence on water quality in the canal. Atmospheric N and P deposition and meteorological factors accounted for 18% of the water quality. The contribution rate and ecological impact of atmospheric N and P deposition on water pollution of main channels will be increasing, which should be paid enough attention to. According to the results of this research, the appropriate location and period could be chosen for water protection and restoration, which could ensure the safety of the SNWD water supply. The study could also provide the data basis for the management of the Hebei and Tianjin sections of the SNWD main canal.

## Figures and Tables

**Table 1:** Locations of sampling stations in the MRP of the SNWD in Henan Province.

City/Country	Station	Code	Population(w)	Longitude	Latitude
Taocha Country	Taocha	TC	0.070	111.705	32.676
Nanyang City	Nanyang	NY	962.90	112.461	32.999
Yexian City	Yexian	YX	73.40	113.137	33.529
Jiaxian City	Jiaxian	JX	50.64	113.227	34.006
Yuzhou City	Yuzhou	YZ	126.98	113.503	34.186
Zhengzhou City	Zhengzhou	ZZ	1274.20	113.558	34.747
Jiaozuo City	Jiaozuo	JZ	352.30	113.211	35.216
Weihui City	Weihui	WH	48.59	114.328	35.919
Hebi City	Hebi	HB	157.20	114.256	35.744
Anyang City	Anyang	AY	542.30	114.234	36.096

**Table 2:** Physical and chemical characteristics of water quality in different locations of the MRP in Henan Province.

Stations	AY	HB	JX	JZ	NY	TC	WH	YX	YZ	ZZ
pH	8.78 ± 0.23	8.75 ± 0.24	8.69 ± 0.21	8.75 ± 0.22	8.63 ± 0.19	8.65 ± 0.42	8.76 ± 0.23	8.68 ± 0.2	8.71 ± 0.22	8.73 ± 0.21
DO	8.65 ± 1.40	8.54 ± 1.27	8.37 ± 1.20	8.54 ± 0.95	8.29 ± 0.97	8.35 ± 1.3	8.37 ± 1.13	8.07 ± 0.99	8.31 ± 1.15	8.4 ± 1.04
Cond	246.83 ± 34.45	245.27 ± 30.00	251.54 ± 28.12	249.10 ± 31.87	254.81 ± 27.08	249.23 ± 25.65	246.76 ± 31.38	255.53 ± 27.76	252.68 ± 29.85	248.5 ± 27.55
ORP	152.46 ± 70.63	152.29 ± 69.12	150.69 ± 75.30	157.52 ± 65.82	147.30 ± 76.65	146.79 ± 52.4	155.78 ± 72.13	155.52 ± 69.87	158.81 ± 66.24	165.94 ± 65.16
COD	10.57 ± 2.48	11.27 ± 2.79	11.20 ± 3.26	11.80 ± 2.59	9.90 ± 2.50	9.92 ± 2.68	10.92 ± 2.66	9.84 ± 2.92	11.07 ± 2.42	11.21 ± 2.23
COD_Mn_	2.58 ± 0.70 ab	2.58 ± 0.70 ab	2.87 ± 0.91 a	2.48 ± 0.72 ab	2.39 ± 0.56 ab	2.34 ± 0.74 ab	2.73 ± 0.66 b	2.53 ± 0.62 ab	2.6 ± 0.75 ab	2.44 ± 0.7 ab
TOC	4.28 ± 1.22 b	4.37 ± 0.91 ab	4.54 ± 1.53 ab	4.87 ± 2.12 ab	4.56 ± 1.25 ab	4.08 ± 1.45 b	4.65 ± 1.54 ab	4.19 ± 0.93 b	4.51 ± 1.64 ab	5.25 ± 1.68 a
TP	0.01 ± 0.01 b	0.01 ± 0.01 b	0.02 ± 0.01 ab	0.02 ± 0.01 ab	0.02 ± 0.01 ab	0.02 ± 0.01 ab	0.02 ± 0.01 ab	0.02 ± 0.01 ab	0.02 ± 0.01 a	0.02 ± 0.01 ab
TN	1.38 ± 0.31	1.42 ± 0.34	1.54 ± 0.36	1.4 ± 0.40	1.52 ± 0.39	1.52 ± 0.46	1.43 ± 0.36	1.45 ± 0.34	1.4 ± 0.22	1.40 ± 0.36
NH_4_^+^-N	0.08 ± 0.06	0.11 ± 0.10	0.10 ± 0.08	0.11 ± 0.11	0.09 ± 0.06	0.09 ± 0.05	0.13 ± 0.13	0.12 ± 0.12	0.13 ± 0.13	0.10 ± 0.09
NO_3_^−^-N	0.87 ± 0.23	0.86 ± 0.23	0.90 ± 0.22	0.90 ± 0.28	0.95 ± 0.19	0.95 ± 0.16	0.88 ± 0.28	0.86 ± 0.24	0.84 ± 0.24	0.85 ± 0.28
chla	2.29 ± 2.26	2.07 ± 1.91	1.64 ± 0.81	1.65 ± 1.26	1.60 ± 0.99	1.66 ± 1.32	1.96 ± 1.58	1.58 ± 0.86	1.68 ± 1.07	1.74 ± 1.40
Water Quality Classification	II	II	II	II	II	II	II	II	II	II

Note: Different lowercase letters in the same line indicate different groups, with significant differences between different groups (*p* < 0.05) in different seasons.

**Table 3:** Seasonal variations of meteorological factors and water quality factors.

	Season	2019 Dry	2019 Wet	2020 Dry	2020 Wet
Meteorologic	AT (°C)	6.08 ± 4.21 b	30.34 ± 9.54 a	8.78 ± 6.12 b	31.98 ± 6.03 a
	AP (hPa)	1024.50 ± 10.17 a	995.65 ± 13.16 c	1014.90 ± 5.80 b	992.93 ± 5.93 c
	AH (%)	44.17 ± 15.95 b	49.59 ± 12.68 ab	45.62 ± 24.32 b	55.51 ± 17.47 a
	WS (m/s)	1.33 ± 15.95 b	1.92 ± 0.97 a	1.44 ± 0.58 b	1.03 ± 0.92 b
	PM_2.5_ (μg/m^3^)	85.30 ± 37.70 a	44 ± 9.50 c	54.92 ± 32.77 b	14.74 ± 11.02 d
	PM_10_ (μg/m^3^)	95.23 ± 42.09 a	50.26 ± 10.32 b	61.50 ± 36.79 b	16.57 ± 12.58 c
Water quality	pH	8.60 ± 0.13 a	8.67 ± 0.23 a	6.051 ± 4.03 b	8.83 ± 0.21 a
	DO (mg/L)	8.40 ± 0.37 a	7.85 ± 0.94 a	7.40 ± 5.07 a	8.21 ± 0.98 a
	Cond (μS/cm)	238.57 ± 14.70 a	283.55 ± 16.80 a	126.78 ± 92.42 b	253.25 ± 12.15 a
	ORP (mv)	193.87 ± 34.49 a	175.21 ± 51.21 a	92.99 ± 72.68 b	110.10 ± 27.98 b
	COD_Mn_ (mg/L)	1.86 ± 0.26 b	2.85 ± 0.64 a	2.58 ± 0.64 a	2.76 ± 0.75 a
	TOC (mg/L)	5.58 ± 1.19 a	4.13 ± 1.30 ab	3.68 ± 1.03 a	4.77 ± 2.17 b
	TP (mg/L)	0.02 ± 0.009 b	0.02 ± 0.008 a	0.01 ± 0.01 b	0.017 ± 0.006 ab
	TN (mg/L)	1.31 ± 0.28 b	1.48 ± 0.34 a	1.48 ± 0.17 a	1.59 ± 0.33 a
	NH_4_^+^-N (mg/L)	0.03 ± 0.03 c	0.12 ± 0.05 ab	0.09 ± 0.12 b	0.15 ± 0.12 a
	NO_3_^−^-N (mg/L)	0.75 ± 0.23 a	0.89 ± 0.20 b	1.09 ± 0.19 a	0.93 ± 0.14 b
	Chla (mg/m^3^)	0.7 ± 0.42 c	1.80 ± 1.17 b	1.17 ± 0.95 c	3.08 ± 1.9 a

Note: Different lowercase letters in the same line indicate different groups, with significant differences between different groups (*p* < 0.05) in different seasons.

## Data Availability

Not applicable.
